# The Brain Reaction to Viewing Faces of Opposite- and Same-Sex Romantic Partners

**DOI:** 10.1371/journal.pone.0015802

**Published:** 2010-12-31

**Authors:** Semir Zeki, John Paul Romaya

**Affiliations:** Wellcome Laboratory of Neurobiology, University College London, London, United Kingdom; CNRS, France

## Abstract

We pursued our functional magnetic resonance imaging (fMRI) studies of the neural correlates of romantic love in 24 subjects, half of whom were female (6 heterosexual and 6 homosexual) and half male (6 heterosexual and 6 homosexual). We compared the pattern of activity produced in their brains when they viewed the faces of their loved partners with that produced when they viewed the faces of friends of the same sex to whom they were romantically indifferent. The pattern of activation and de-activation was very similar in the brains of males and females, and heterosexuals and homosexuals. We could therefore detect no difference in activation patterns between these groups.

## Introduction

The work reported here is a continuation of our previous work on brain systems and networks that are critical for the sentiment of romantic love [1], [2]. It was inspired by a reading of the world literature of love, both Western and Oriental, in which similar sentiments are expressed, whether in the same or opposite sex context. In extending our work, we therefore considered it interesting to compare the pattern of brain activity evoked in opposite- and same-sex lovers when they view the pictures of those they love. Passionate romantic love, commonly triggered by a visual input, is an all-consuming and disorienting state that pervades almost every aspect of a lover's life. Yet human brain imaging studies [1], [2], [3], [4] show that the neural correlates of viewing the face of a loved person are limited to only a few, though richly connected, brain regions. This limitation made it plausible to suppose that we could detect any differences relatively easily. Differences between homosexual and heterosexual brains have been described, specifically in the size of hypothalamic [5] or suprachiasmatic [6] nuclei, or in the degree of lateralization between the two groups of men [7], or in hemispheric asymmetries and differential activation patterns between homosexual and heterosexual brains. But such differential activations as have been described have been in response to sexually arousing stimuli [8], not in response to the sentiment of love. Given the profound similarity in the sentiment of love expressed in the opposite- or same-sex contexts, we hypothesised that we would see no differences when females or males, or heterosexual or homosexual subjects, viewed the face of their loved partners. This would amount to a negative result but one that is nevertheless of considerable significance.

## Materials and Methods

### Ethics Statement

Informed written consent was obtained from all participants and the study was approved by the University College London Research Ethics Committee.

### Subjects

28 healthy subjects (equally divided between male and female and heterosexual and homosexual) were recruited through advertisements requesting volunteers who were passionately in love. All reported being in a sexual relationship with their lover. Their age varied from 19 to 47 years (mean 26.3 , ssd 6.4) and length of relationship from 4 months to 23 years (mean 3.7, ssd 4.4). Two subjects were left handed. Subjects were drawn from West European, East European, American, Oriental and Asian backgrounds, within which there were further cultural sub-groupings, for example, British, Italian, Portuguese, etc… within the West European grouping.

Of the 28 subjects who were scanned, 4 were excluded for the following reasons: one showed strong artefacts in her scanned image, another subsequently reported deep underlying problems in the relationship, a third fell asleep shortly after scanning commenced and the fourth subsequently reported thinking of her lover throughout the scanning session, even when neutral faces were being displayed. Later analysis of the individual results from these four excluded subjects displayed very little or no activation for the contrast *Loved vs. Neutral*.

During a first visit to the laboratory, some two weeks prior to scanning, each subject provided 6–8 picture portraits of their lover and a similar number of portraits of other friends of the same sex as their lover towards whom they had neutral feelings, all pictures being matched as far as possible for expression and general appearance. The experiment was explained to the subject and an example stimulus using random anonymous faces was demonstrated. Each subject completed a Passionate Love Scale (PLS) [9] questionnaire, to attempt to quantify their feelings about their lover. Age and length of relationship were recorded for each subject.

During scanning sessions subjects' heart-rate and respiration were continuously recorded, providing physiological measurements that were subsequently incorporated into the first level analysis for each subject (see File S1: *Physiological noise correction*). We also recorded eye gaze position to monitor subjects' attention and galvanic skin response (GSR) but did not use these measurements in the analysis since in this, as in our past studies, we have found the GSR to be an unpredictable and unreliable metric [1]. Directly after scanning, each subject again completed the PLS questionnaire, in order to re-quantify their feelings immediately after the scanning. Subjects were also interviewed to assess whether they had experienced any difficulties (such as that experienced by the excluded subject who reported thinking of her lover throughout the experiment). Subsequent to the experiments, each subject also gave a Kinsey rating of their sexual orientation, on a scale of 0 (exclusively heterosexual) to 6 (exclusively homosexual) [10] (see Table 1). Of the 24 subjects, 50% were exclusively either heterosexual or homosexual. The remaining 50%, whose ratings fell in between, nevertheless declared their relationship to be either heterosexual or homosexual.

**Table 1:** Kinsey ratings of our sample of subjects, ranging from 0 (exclusively heterosexual), through 3 (equally heterosexual and homosexual) to 6 (exclusively homosexual).

	Kinsey rating						
	0	1	2	3	4	5	6
Male heterosexual	4	2	-	-	-	-	-
Male homosexual	-	-	-	-	-	2	4
Female heterosexual	3	2	1	-	-	-	-
Female homosexual	-	-	-	1	-	4	1

### Stimuli

**Figure 1:**
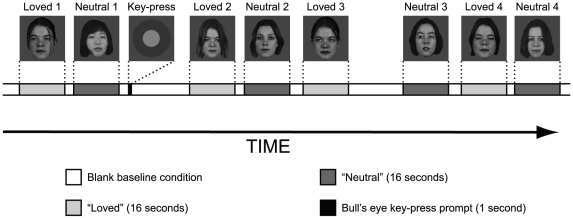
Schematic diagram showing a short typical stimulus subsequence.

Stimuli were generated using Cogent 2000 and Cogent Graphics (http://www.vislab.ucl.ac.uk/cogent.php). Photographic images provided by each subject were digitized, converted to grayscale and edited to remove superfluous features such as earrings, scarves etc. Background detail was replaced with a flat mid-grey tone and images were normalized in terms of visual area and average brightness. Spatial frequency and contrast were also roughly normalized (see File S1: *Preprocessing of face images*). Subjects were exposed to two stimulus sessions but in two subjects the second session was not used, for the following reasons: one subject fell asleep during the second session while for the other the second session was invalid due to technical reasons. The session began with a flat grey background (intensity 6.4 cd/m^2^) (blank condition) which was present for 26 s, during which the first six brain volumes were discarded to allow T1 equilibration effects to subside. The stimulus sequence then began. We used a conventional block design with 16 s epochs during which either a loved or a neutral face was shown. There were 15 epochs of each sort of face (*Loved* and *Neutral* conditions) and 15 baseline blank epochs (*Baseline* condition), with a randomly jittered (0.25 to 0.75 s) blank period between epochs. The 45 epochs were presented in a pseudo-random sequence but no two sequential epochs were of the same type (i.e. *Loved - Loved* did not occur). Since there was a limited number of face images available, they were repeated in a pseudo-randomized sequence through the epochs. Subjects were allowed to scan the images freely and eye movement was recorded for all but three subjects (because of practical difficulties with eye tracking). Interspersed randomly through the sequence of epochs were twelve key-press prompts. To ensure consistent attention over time (and between subjects) participants were required to press a key when a circular bulls-eye prompt appeared for 1 s. For one of the subjects a longer sequence was used (54 epochs and 15 key-press events) but the sequencing and timing (16 s epochs) were similar to the other 23 subjects. The session ended with a blank period of 30 s, during which the scanner continued to acquire decaying BOLD signal. A block design incorporating null events with *ca.* 16 s epochs was chosen for direct comparison with our previous studies on romantic love, maternal love and hate [1], [2], [11] (see Figure 1). To maintain confidentiality the example faces used in Figure 1 are from the XM2VTSbd database [12] (http://www.ee.surrey.ac.uk/Research/VSSP/xm2vtsdb), not from our subjects.

The faces presented and the key-press prompt all had the same average intensity as the blank condition (6.4 cd/m^2^). For each subject, all the faces and the key-press prompts subtended the same visual extent, although this varied between subjects from 0.013 to 0.030 steradians, because of differences in viewing geometry between various subjects. In general, the images subtended a visual angle of about 10°.

### Scanning details

Scans were acquired using a 1.5-T Siemens Magneton Sonata MRI scanner fitted with a head volume coil (Siemens, Erlangen, Germany) to which an angled mirror was attached, allowing subjects to view a screen onto which stimuli were projected using an LCD projector. An echo-planar imaging (EPI) sequence was applied for functional scans, measuring BOLD signals (echo time TE = 50 ms, repeat time TR = 90 ms, volume time 4.32 s). Each brain image was acquired in a descending sequence comprising 48 axial slices each 2 mm thick with an interstitial gap of 1 mm and a voxel resolution of 3 mm, covering nearly the whole brain. BOLD sensitivity losses in the amygdala due to susceptibility artefacts were minimized by optimizing z-shim gradient moment, slice tilt and PE gradient polarity [13]. After functional scanning had been completed a T1 MDEFT anatomical scan was acquired in the sagittal plane to obtain a high resolution structural image (176 slices per volume, constant isotropic resolution of 1 mm, TE = 3.56 s, TR = 12.24 s).

### Analysis

Data were analyzed using SPM5 (http://www.fil.ion.ucl.ac.uk/SPM) [14]. The time series of functional brain volume images for each subject was realigned and normalized into MNI (Montreal Neurological Institute) space (voxel size 3×3×3 mm) and then smoothed using a Gaussian smoothing kernel of 9 mm (FWHM). The stimulus was a block design and the onsets and durations of the appearances of the loved and neutral faces were modeled as boxcar functions. Key-presses were separately modeled as delta functions. Head movement parameters calculated from the realignment pre-processing step and physiological data acquired during the scan (heart-rate and respiration) were also included in the model (see File S1: *Physiological noise correction*). Stimulus functions were convolved with the default SPM5 canonical hemodynamic response function and entered into a linear convolution model (for each subject). Maximum likelihood estimates of the associated parameters were then taken to the second (between subject) level for random effects inference. This involved taking contrasts or mixtures of parameter estimates summarizing condition-specific effects in each subject and creating SPMs of unpaired t-statistics using these contrast images.

## Results

We only report cluster activations that were significant at p<0.05 corrected for the whole brain. In addition, we report clusters that were *trend significant* (denoted in italics), at p<0.10 corrected for the whole brain. Where we expected activity from previous publications, we used a small search volume of 16 mm radius (denoted p_SVC r16_), centred on the previously cited co-ordinate.

We were interested in the effects *Loved > Neutral* and *Neutral >Loved* across all subjects.

### Loved>Neutral

**Figure 2:**
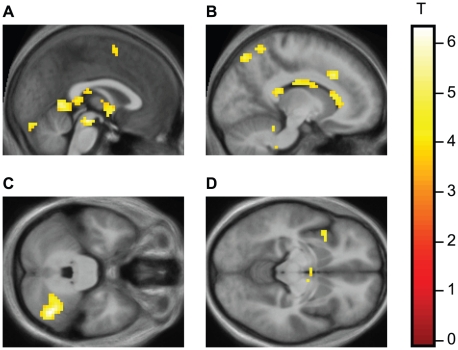
Illustration of the t statistic for the contrast *Loved > Neutral* showing selected activations superimposed over averaged anatomical sections (the average of the 24 subjects in our sample). Random effects analysis with 24 subjects. Background threshold p_uncorr_<0.001. Cluster threshold k_E_> = 10. (A) Medial sagittal plane (x = 0) showing activations in the tegmentum, hypothalamus and vermis. (B) Sagittal plane x = −12 (LH) showing activation in the caudate head, anterior cingulate and parietal cortex. (C) Horizontal plane z = −30; right cerebellum. (D) Horizontal plane z = −9; mid insula, left hemisphere.

There were eight areas of activation significant at the cluster level in addition to three areas that were trend significant (see Table 2). It is notable that the activity in the caudate nucleus was not restricted to one locus but distributed in clusters over the head and the body (see Figure 2).

**Table 2:** Activations for the contrast *Loved>Neutral* (Activations with loved faces).

	x	y	z	k_E_	p _Clust._
Hippocampus LH	−21	−39	15	316	5.5×10^−8^
Hippocampus RH	33	−36	0	44^1^	0.001
Caudate head LH	−18	−15	24	25^1^	0.007
Caudate head RH	15	−9	24	200	8.6×10^−6^
Cerebellum (Crus 1) RH	42	−63	−30	142	1.6×10^−4^
Hypothalamus	−3	−3	−6	92	0.003
Vermis	0	−51	3	64	0.016
Superior parietal lobule LH	−21	−51	51	59	0.023
*Tegmentum*	*0*	*−24*	*−15*	*47*	*0.054*
*Anterior cingulate LH*	*−12*	*21*	*33*	*41*	*0.086*
*Caudate nucleus (Body) LH*	*−9*	*24*	*9*	*40*	*0.093*

All activations are cluster significant at p<0.05 (corrected) or *trend significant* (indicated in italics) at p<0.10 (corrected). Random effects analysis with 24 subjects. Clusters are thresholded at a background level of p_uncorr._<0.001 unless the cluster size k is superscripted ^1^ in which case the background threshold was lowered to p_uncorr._<0.0001 to isolate sub-clusters within a larger group. Cluster probabilities were calculated using random field theory. Under the null hypothesis the expected cluster size was 4.9 for a background threshold of p_uncorr._<0.001 or 2.4 for a background threshold of p_uncorr._<0.001.

The medial insula (not shown in Table 2) and strongly active at [−44, 6, −4] in our previous study [1], became evident when using a small search volume in the left hemisphere at [−39, 12, −9] (p_SVC r16_ = 0.036).

### Neutral>Loved

**Figure 3:**
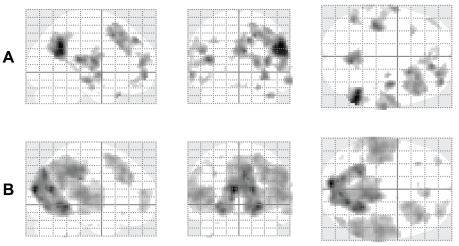
SPM maximum intensity projection (MIP) of the t statistic for the contrasts *Neutral > Loved* (A) and *Baseline>Loved* (B) obtained from a random effects analysis with 24 subjects. Background threshold p_uncorr._<0.001.

The overall pattern obtained for this contrast was very similar to the one described in our previous study [1]. It was widely distributed and included the frontal, parietal and temporal cortex, medially and laterally (Figure 3 A and Table 3). The amygdaloid region, appearing as a de-activation in [1] at [22, −8, −22], was also evident in this study at [24, −12, −21] (p_SVC r16_ = 0.018).

**Table 3:** Activations for the contrast *Neutral>Loved* (De-activations with loved faces).

	x	y	z	k_E_	p _Clust._	t	Z	p _FWE-corr._
Superior frontal gyrus RH	21	15	48	469	1.7×10^−10^	*6.34*	*4.64*	*0.096*
Parietal cortex (BA39) RH	54	−54	33	419	1.0×10^−9^	9.30	5.72	3.0×10^−4^
Mid. Tmp. Gyr. (BA21/22) RH	66	−18	−12	267	4.2×10^−7^			
Middle orbital gyrus RH	33	57	−6	178	2.5×10^−5^			
Rolandic operculum RH	60	−6	15	52^1^	1.9×10^−4^			
Rolandic operculum LH	−39	−27	15	205	6.8×10^−6^	6.94	4.90	0.027
Precuneus RH	6	−60	27	246	1.1×10^−6^	6.86	4.87	0.032
Superior frontal gyrus RH	24	54	12	37^1^	0.001			
Superior frontal gyrus RH	12	45	42	113	0.001			
Ang. Gyr. (Parietal cortex) LH	−48	−57	27	101	0.002			
Insular lobe RH	36	−9	15	68	0.012	*6.43*	*4.68*	*0.081*
*Superior temporal gyrus LH*	*−63*	*−3*	*−3*	*44*	*0.068*			

All activations are cluster significant at p<0.05 (corrected) or *trend significant* (indicated in italics) at p<0.10 (corrected). Random effects analysis with 24 subjects. Clusters are thresholded at a background level of p_uncorr._<0.001 unless the cluster size k_E_ is superscripted ^1^ in which case the background threshold was lowered to p_uncorr._<0.0001 to isolate sub-clusters within a larger group. Cluster probabilities were calculated using random field theory. Under the null hypothesis the expected cluster size was 4.9 for a background threshold of p_uncorr._<0.001 or 2.4 for a background threshold of p_uncorr._<0.001. Some of these locations were also significant at the peak level corrected for familywise error over the whole brain, indicated as p _FWE-corr._ and these are indicated by entries in the three rightmost columns.

### Baseline>Loved

**Figure 4:**
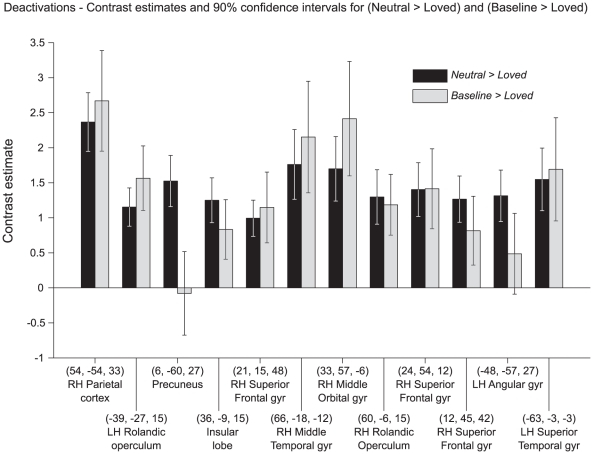
De-activations with love. Contrast estimates for *Neutral > Loved* at the locations listed as significant in Table 3 are shown in black. At each of these locations the corresponding contrast estimate for *Baseline > Loved* is shown in grey.

To learn whether the contrast *Neutral > Loved* reflects a true pattern of de-activation or whether it only reveals a diminished activity for loved faces compared to neutral ones, we looked at the contrast *Baseline > Loved* (where the baseline condition was a flat grey, featureless background – see Methods). This latter contrast should reveal whether there are any regions in the brain where activity is suppressed when viewing a lover's face relative to baseline conditions. The resulting pattern, shown in Figure 3B, was similar to that of Figure 3A. Apart from a de-activated visual cortex (see below), the pattern was very similar in the contrasts Neutral > Loved and Baseline > Loved. Hence every locus that was de-activated in the Neutral > Loved was also de-activated in the Baseline > Loved, with the exception of the precuneus. The de-activation in visual cortex obtained in the contrast Baseline > Loved has an antecedent in the observations of Smith et al. [15], who described widespread deactivation of those parts of the visual cortex which are outside the focus of attention in conditions when observers attend to particular locations in the visual field. The histogram of Figure 4 compares the contrast estimates for *Neutral > Loved* and *Baseline > Loved*. It also shows that at every location, except the precuneus, where there was de-activation in the former, there was also de-activation in the latter. We conclude that the contrast *Neutral > Loved* shows a genuine de-activation.

### Putamen

The dorsal putamen, bilaterally but weakly active in our previous study [1] at [−22, 0, 10] and [26, 0, 2], was not apparent in the contrast *Loved > Neutral* in this study. However, inspection of the contrasts *Loved > Baseline* and *Neutral > Baseline* revealed a complex pattern of activity here. In some specific locations there was activity for the contrast *Loved > Baseline* which reached significance but which was cancelled out by a corresponding, weaker, sub-threshold activation for the contrast *Neutral > Baseline*. Thus, the dorsal putamen was significantly active for the contrast *Loved > Baseline* at [−24, 6, 6] (p_SVC r16_ = 0.038) and [21], [3], [9] (p_SVC r16_ = 0.014). Elsewhere, a peak in the right putamen at [24, −12, 12] was significant for *Neutral > Baseline* (p_FWE-corr._ = 0.025) but this was cancelled out by an activation at that location for *Loved > Baseline* and so was not significant for the contrast *Neutral > Loved*. The contrast estimates at these locations are detailed in File S1: *Putamen activations*. Thus the putamen was active for both loved and neutral faces.

### Main effects of gender and sexual orientation

The second level random effects analysis considered the contrast *Loved vs. Neutral* with the subjects grouped according to the factors of gender (male or female) and declared sexual orientation (heterosexual or homosexual) in a 2×2 factorial design. Thus there were four groups; 6 male heterosexuals, 6 male homosexuals, 6 female heterosexuals and 6 female homosexuals. With the data so grouped by gender and declared sexual orientation we now tested for the effects of these factors by examining the contrasts *Male vs Female* and *Heterosexual vs Homosexual* respectively. No significant effects were found for either factor. In other words, in our sample of 24 subjects, differences between the sub-groups (male/female, heterosexual/homosexual) were not significantly greater than the overall differences within those subgroups. We thus did not observe any significant effect within our sample between males and females or between heterosexuals and homosexuals.

### Interaction between gender and sexual orientation

In the absence of significant main effects we next considered the interaction between gender and sexual orientation for *Loved vs Neutral*. In this 2×2 between-subjects design the interaction is represented by the contrast *Attracted to Females vs Attracted to Males* (where the group *Attracted to Females* consists of the two subgroups, heterosexual males and homosexual females, and the group *Attracted to Males* consists of the two subgroups, heterosexual females and homosexual males) [16]. No significant interaction was found in our sample.

In a further exploration, perhaps of limited value given the small sample size of six subjects per sub-group and despite the lack of significant interaction, we considered four further contrasts; *Heterosexual Males vs Rest*, *Homosexual Males vs Rest*, *Heterosexual Females vs Rest* and *Homosexual Females vs Rest*, where *Rest* consisted of the three remaining sub-groups in each case. None of these four contrasts yielded any significant activations or deactivations, either at the peak or cluster levels.

### Covariation with PLS, subject age and relationship length

We collected each subject's PLS score (which could range from 0 to 120). The scores in our sample ranged from 61 to 117, with a mean of 100.1. We also recorded subject age (range 19 to 47 years, mean 26.3 years) and length of relationship with their lover (range 4 months to 23 years, mean 3.7 years). We supposed that effects of *Loved vs Neutral* might covary with one or more of these parameters, especially since such covariation has been reported by Aron et al. [3]. We found that these three parameters displayed some degree of correlation, in that older subjects had been in longer relationships and also scored less on the PLS. For this reason we analyzed each of these parameters separately. For these analyses we were not concerned with differences due to gender or sexual orientation; therefore data for all 24 subjects was combined into a single group at the second level analysis, with a single covariate - either PLS, subject age or relationship length.

No significant correlations were found for PLS score or subject age.

We found a significant negative correlation between *Loved > Neutral* and relationship length in the full set of 24 subjects at three locations (see File S1: *Correlations with relationship length*). However, one subject had had a much longer relationship (23 years) than the others; when he was excluded no significant correlation was found, suggesting either that such a correlation may only be detectable over longer timespans or that this one subject may represent an anomalous outlier.

## Discussion

The main aim of this continuation of our studies on love was to determine whether there are any differences in the pattern of brain activity between males and females and heterosexuals and homosexuals when they view pictures of those they love, which amounts to enquiring whether there is any difference between male and female or heterosexual and homosexual brain patterns in response to romantic love. Since our results have shown no differences in the pattern of brain activation produced in these different groups, the discussion below applies to all. As well, since the pattern of activation obtained here is very similar to our previous results [1], [2], we will not discuss the significance of each active site, which we have done in our previous papers. Instead, we restrict ourselves to discussing the differences between patterns of activation obtained in this and previous studies and to discussing the results against the background of the world literature of love, which provided the inspiration for this study.

We begin by emphasizing that any study of so complex and overpowering a sentiment as love is fraught with difficulties. Chief among these is that the sentiment itself involves many components – erotic, emotional, and cognitive – that are almost impossible to isolate from the overall sentiment of love. The converse is not true, in that a component such as the erotic can be independent of love and independently studied, as has been done in recent studies [8], [17], [18], [19], [20]. While acknowledging this difficulty, we tried as best we could to circumvent it, by applying a uniform criterion – that of a loved face – for studying the brain's love system. Another problem is the difficulty of controlling the mental processes that occur when subjects view their lovers' faces. The only way to address this is through the statistical methods we have used to analyze our results. We have employed a random effects analysis using the summary statistic approach [21] to control for the between-subject variation in our sample. This enables us to extract what is common to the subjects and to infer the stereotypical effect in the wider population from which our sample is drawn.

As commonly reported by those who have written about love in world literature, it is the visual input, especially that of a face, that is the most potent in arousing it. And since a critical part of our inspiration for this study is drawn from that literature (*see below*), it is as well to exemplify it by Dante's lines in the *Paradiso*, celebrating the life-long romantic infatuation with Beatrice triggered by his first view of her face:

“From the first day that I saw her face

in this life, to this very moment

the sequence of my song has never ceased”

Hence, to be precise, the results we report here describe the brain reaction to viewing the face of a loved partner, which opens a window into understanding a little about the brain's love system.

### Loved > Neutral

Confirming earlier studies by us [1], [2] and others [3], [4], the pattern of activation produced by our paradigm can be said to have a core with extensions into the cerebral cortex and the cerebellum. The core, consisting of the basal ganglia (caudate and putamen), the ventral tegmental area (VTA), and the hypothalamus, is rich in dopaminergic inputs. Dopamine (a neurotransmitter linked to the motivational state of “wanting”) has been shown to be important in a variety of contexts, among them several that are important for this study, namely reward and its expectation [22], mood [23], “wanting” [24], motivation [25] and emotional memory [26]. It is produced in a number of areas, prominent among them two that were active in this study, namely the hypothalamus and the VTA. Beside the basal ganglia, the dopaminergic system projects widely to the cerebral cortex, cerebellum and hippocampus [27]. Dopamine is intimately linked to other neurohormones that have been implicated in romantic relationships. They include oxytocin, vasopressin (both synthesised in the hypothalamus) [28], serotonin [29] (also present in the hypothalamus) and norepinephrine [23], [30]. These are also richly distributed in areas that were active in the present study – the hypothalamus, VTA and the caudate nucleus.

The cerebellum, active in this study, was also active in our previous study [1], though not commented on. Traditionally regarded as a motor centre, it has been shown to play a role in emotional conditions, especially the recall of emotional memories and empathy with a lover [31]. The cerebellar vermis, known to have dopaminergic input, may be involved in reward-related activities [32] and also play a role in craving, since it has been shown to be active during states of thirst [33].

Dorsal hippocampus, also not commented on in our previous study, differs in its connections from ventral (or anterior) hippocampus, and is thought to perform primarily cognitive functions [34]. But it has also been reported to exert strong regulatory control of the hypothalamic-pituitary-adrenal axis and hence presumably on the neurohormonal and neurotransmitter systems that are critical in pair-bonding and love relationships. Decreased hippocampal volumes and hippocampal dysfunction are associated with psychological disorders with strong affective components such as post-traumatic stress syndrome, bipolar disorder and depression [34].

### Neutral > Loved

Figure 3 and Table 3 show that the results obtained for this contrast are very similar to those obtained in our previous studies [1], [2], and include frontal, parietal and temporal cortex. We could detect no significant differences between males and females or between heterosexuals and homosexuals for this contrast. We have previously commented on the significance of this picture, which we referred to as a de-activation [1]. The present results for the contrasts *Neutral > Loved* and *Baseline > Loved* (Figures 3 and 4) show that there is indeed a pattern of cortical de-activation which includes large areas of cortex, involved in a variety of different functions, including judgmental ones.

### Comparison to other studies

In showing activation of brain regions that are rich in neurohormones implicated in emotional states and pair-bonding, the results we report here are in broad agreement with our own previous results [1], [2] as well as with those of Aron et al. [3]. There are however differences that are worth highlighting. Our studies of maternal and romantic love led to activation of the cerebellum and parietal cortex as well as the hippocampus while these areas have not been reported to be active in the study of Aron et al. [3]. As well, there is a difference in the pattern of de-activation (obtained from the contrast *Neutral > Loved* in our studies) and the pattern of de-activation in the study of Aron et al. [3]. While in the latter the de-activation was restricted to the amygdala, the de-activation we obtained in our previous studies [1], [2], as well as in this one, went beyond and involved very large regions of the brain, extending from parietal to frontal and temporal cortex. The reason for this difference is not obvious. It may lie in variations in the paradigm used (they used a countback between positive and neutral stimuli to provide a distraction, whereas we did not). It may also lie in the length of relationship, with ours being on the whole longer than theirs. But this latter reason would not account for another difference between our results and theirs, namely our failure to find any correlation between the PLS, length of relationship and activation intensity in any of the active sites, while they reported such a relationship for insula and caudate. The only possible relationship that may exist in our results is due to one subject, who reported a relationship lasting 23 years. Thus, it is possible that a significant difference becomes apparent only with relatively long periods of romantic attachment. In a sense, our failure to find differences, especially one relating to the length of relationships, is surprising since a decline in the intensity of passion with time is a common experience and has been documented [35]. We currently have no way of accounting for these differences, which will no doubt be resolved in future studies.

### No detectable differences with respect to gender or sexual orientation

The main purpose of this study was, however, to learn whether there is any difference in the pattern of activation between heterosexuals and homosexuals in viewing the pictures of partners to whom they are romantically attached. We wanted to address the question because of previous reports of structural differences between heterosexual and homosexual brains [5] [6], or in hemispheric lateralization [7], or asymmetries and differential activation patterns between homosexual and heterosexual brains in response to sexually arousing stimuli [8]. In spite of this, we could not detect any differences related to either gender or sexual orientation, either through an analysis of the main effects or of their interaction.

That essentially the same brain areas should be active in heterosexual and homosexual subjects, regardless of sexual orientation, during the experience of love triggered by viewing the face of a loved person, should perhaps occasion no surprise. The world literature of love is very uniform in this regard, whether Western or Oriental or whether expressed in the same or opposite sex context. Central to it are two themes – the desire to be united with the lover and to be annihilated with, and in, the lover [36]. They are forcefully there in Wagner's *Tristan und Isolde*, in the Farsi poetry of Rumi and Hafiz, the Arabic-Azeri legend of Majnun and Leila, the *Rime* of Michelangelo, the double suicide *shinju* tradition in Japan as exemplified by the work of Chikamatsu and others, the Hindu legend of Radha-Krishna, the *fana'* (annihilation) of Sufi love literature and much else besides. Indeed, the sentiments expressed are so similar as to introduce a profound ambiguity that makes it easy to read these texts in the opposite- or same-sex contexts, regardless of the authors' intentions. This is true of the sonnets of Shakespeare, among others, and is much aided where the language used is silent as to gender, as in the poetry of Rumi and Hafiz in Farsi. It would have been surprising if this similarity were not reflected somehow in brain activity. Here we have shown that, with the methods currently available to us and using perhaps overly conservative criteria, we could not detect any differences relating to the expression of the sentiment of love in the same or opposite sex context, either in the areas activated or in the intensity of activation within them.

This is of course not to say that differences do not exist, which is indeed implicit in the very classification of lovers into two groups according to orientation. Yet these differences are perhaps best sought elsewhere than in the experience of the sentiment of love when viewing the face of a loved partner, and a challenge for the future lies more in determining their neurobiological source. Perhaps they are better sought for in the sexual counterpart to love. Recent studies have suggested differences in brain activity between heterosexual and homosexual men resulting from viewing visually erotic stimuli. Hu et al [17] have shown, for example, that in addition to a common circuit, different neural circuits are active during sexual arousal in heterosexual and homosexual men.

Another difference may be in the intensity of activation of common areas. Paul et al. [19] have shown that viewing erotic stimuli corresponding to their sexual orientation activates the hypothalamus in both groups, but viewing those corresponding to an orientation opposite to theirs does not, an observation supported by the demonstration that the strength of activation in different brain areas differs when heterosexuals and homosexuals view clips corresponding to their orientation [20]. A difference in strength of activation is also suggested by the observation that men tend to have greater activation of the hypothalamus than women during the viewing of sexually arousing stimuli [18]. As well, it is possible that had we undertaken a far more detailed study and explored activity in the brains of women at particular follicular phases [37], or lovers who fall only into particular Kinsey groups, between exclusive heterosexuality and exclusive homosexuality, we might have detected differences in the intensity of activity in particular brain regions. Our study was however directed more towards the sentiment of love and, given the high average score we obtained on the PLS questionnaire, it would seem that much more detailed studies – ones that would enquire into follicular cycles, exact sexual status, as well as other cognitive factors, including the detailed past history of lovers - would be required to chart such differences in the sentiment of love between different groups, assuming them to exist at this level. Moreover, had we restricted ourselves to the study of a single cultural and socio-economic group, we might have encountered less variability which might have led to the emergence of significant differences between groups.

We have in the past shown that there is a remarkable similarity, though not identity, in the pattern of brain activation produced by viewing the face of a loved partner and the face of a loved child by the mother [2]. Here, we extend this and show that the similarity in the pattern of brain activation produced by viewing the face of a loved partner, regardless of orientation, is even more striking, with no detectable differences. Perhaps, as La Rochfoucauld wrote in his *Maximes*, “There is only one kind of love but there are a thousand different copies”. The challenge for us lies in detecting what determines these different copies, within and between different groups.

## Supporting Information

File S1 Supplementary detail for the following topics: physiological noise correction, processing of face images, putamen activation and correlations with relationship length.
(DOC)
