# Supplementary material for: The Brain Reaction to Viewing Faces of Opposite- and Same-Sex Romantic Partners
Source: PLoS One. 2010 Dec 31;5(12):e15802. doi: 10.1371/journal.pone.0015802 (PMC3013131; doi:10.1371/journal.pone.0015802)
Supplement: File S1 — Supplementary detail for the following topics: physiological noise correction, processing of face images, putamen activation and correlations with relationship length. (DOC) [file pone.0015802.s001.doc]

**SUPPORTING INFORMATION**

***Physiological noise correction***

During scanning sessions peripheral measurements of subject pulse and breathing were made together with scanner slice synchronisation pulses using the Spike2 data acquisition system (Cambridge Electronic Design Limited, Cambridge UK). The cardiac pulse signal was measured using an MRI compatible pulse oximeter (Model 8600 F0, Nonin Medical, Inc. Plymouth, MN) attached to the subject’s finger. The respiratory signal, thoracic movement, was monitored using a pneumatic belt positioned around the abdomen close to the diaphragm.

 A physiological noise model was constructed to account for artifacts related to cardiac and respiratory phase and changes in respiratory volume using an in-house developed Matlab toolbox [1]. Models for cardiac and respiratory phase and their aliased harmonics were based on RETROICOR [2] and a similar, earlier method. Basis sets of sine and cosine Fourier series components extending to the 5th harmonic (i.e. 5 terms) for the cardiac phase and 3rd harmonic for the respiratory phase were used to model the physiological fluctuations. The model for changes in respiratory volume was based on [3]. This resulted in a total of 17 regressors which were sampled at a reference slice in each image volume to give a set of values for each time point. The resulting regressors were included as confounds in the first level analysis for each subject.

***Preprocessing of face images***

Faces were selected from the images provided by each subject, six to eight of the loved person, and a similar number of people for whom the subject had neutral feelings. The images were either full face or three-quarter profile. The faces were rotated if necessary to align them with the vertical axis. As far as possible the expressions (e.g. smiling, serious etc...) were matched for each subject. The images were digitized and an image-editing program (Adobe® Photoshop® CS2) was used to remove any obscuring articles such as earrings, scarves etc... A square containing just the head was cropped from each image. Each of these square images was then resized to the pixel dimensions of the smallest image, to roughly normalize the spatial frequency of the images. The background detail was then replaced with a flat mid-grey tone.

When the images were finally displayed to the subject some further normalization was imposed:-

i/ The images were converted to greyscale.

ii/ The images were individually scaled so that each face had the same surface area.

iii/ The mean brightness of each face was the same.

iv/ The faces were roughly matched in contrast. The frequency distribution of individual pixel intensities was calculated for each image. The overall intensities of the image were stretched linearly about the mean so that the intensity difference between the 5^th^ and 95^th^ percentiles of the frequency distribution was the same for each image. The contrast was maximised for each set of four images so that somewhere in one of the four images there was either a pixel of the darkest (0.23 cd/m^2^) or of the lightest (34.1 cd/m^2^) possible intensity afforded by the projection system.

In order to perform the intensity and contrast normalisations the gamma profile of the projector used to display the images during the second session was measured using a PHOTORESEARCH® PR®-650 spectra-colorimeter. The normalisation was implemented using custom-written scripts for the MATLAB® (©The MathWorks Inc. ) programming environment.

***Putamen activation***

There was activation in the putamen for the contrasts *Loved > Baseline* and *Neutral > Baseline*, but not for *Loved > Neutral* or *Neutral > Loved*. Figure S1 displays contrast estimates and associated confidence intervals at co-ordinates [-24, 6, 6] and [21, 3, 9] bilaterally in the putamen. These locations were near (within 16 mm) locations [-22, 0, 10] and [26, 0, 2] respectively, reported as being active in our previous study [4]. These were significant (p_SVC r16_ < 0.05) for *Loved > Baseline* but not for *Loved > Neutral* because of sub-threshold activity with accompanying variance for *Neutral > Baseline*.


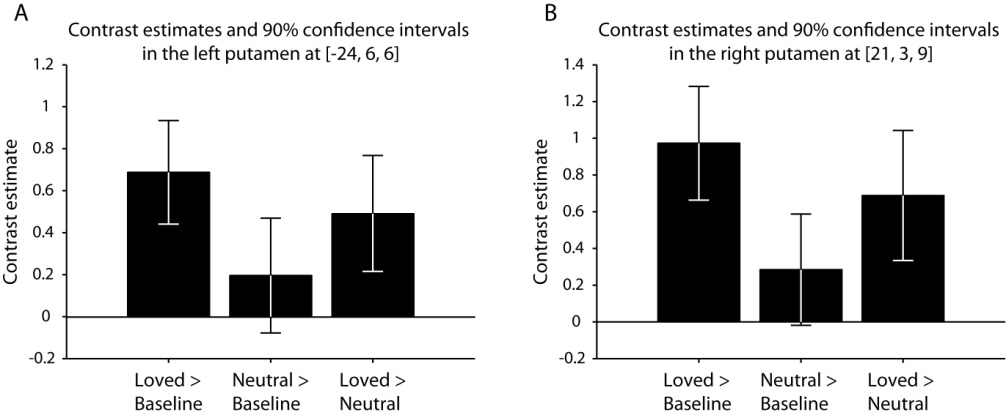


Figure S1 Contrast estimates at locations A [‑24, 6, 6] and B [21, 3, 9], bilaterally in the putamen. Significant (p_SVC r16_ < 0.05) for the contrast *Loved > Baseline* but not for *Loved > Neutral* due to weak activations (with appreciable variance) for the contrast *Neutral > Baseline*.

At another location, in the right putamen at [24, ‑12, 12], there was a significant (p_FWE‑corr._< 0.05) activation for the contrast *Neutral > Baseline*. There was no corresponding significant activity at that voxel for *Loved vs Neutral* due to an activation with high variance there for the contrast *Loved > Baseline* (see Figure S2).


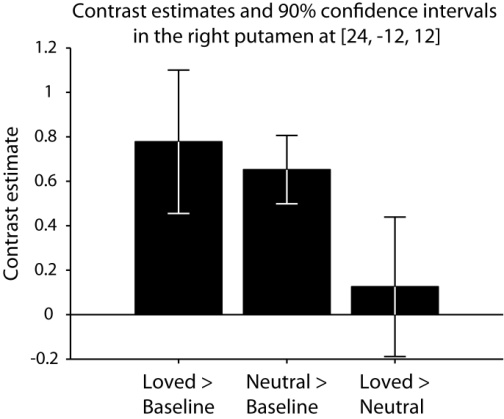


Figure S2 Contrast estimates at [24, ‑12, 12] in the right putamen. Significant (p _FWE-corr_. < 0.05) for the contrast *Neutral > Baseline* but not for *Loved > Neutral* due to activation (with considerable variance) for the contrast *Loved > Baseline*.

***Correlations with relationship length***

We tentatively identify three locations where the contrast *Loved > Neutral* demonstrates a negative correlation with relationship length (Figure S3); in somatosensory cortex at [57, 0, 39] (p _Clust_. = 0.024, k_E_ = 60), on the boundary of the caudate nucleus and the thalamus at [‑3, 0, 9] (p _Clust_. = 0.042, k_E_ = 52) and in parietal cortex at [42, ‑51, 21] (p _Clust_. = 0.022, k_E_ = 61), none of them in the de-activated regions shown in Figure 3.


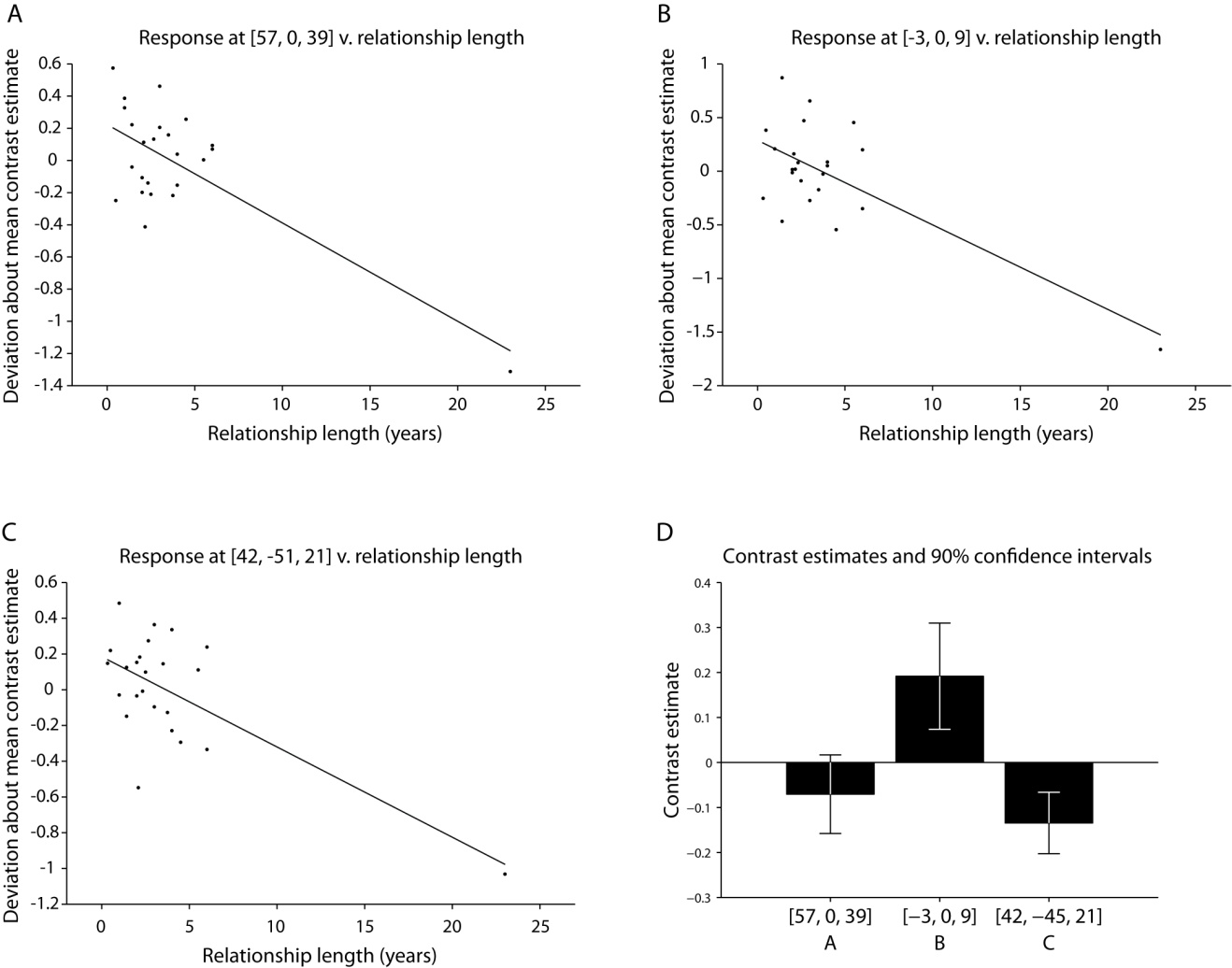


Figure S3 Plots of the contrast estimates for *Loved > Neutral* versus relationship length at three locations where the relationship is significant at the cluster level (p_Clust_. < 0.05); at A [57, 0, 39], B [‑3, 0, 9] and C [42, ‑51, 21]. The contrast estimates for the mean main effect (Loved > Neutral) at these locations are shown in D and are not significantly different from zero (p_FWE-corr_. > 0.10).

It is obvious from the plots that the relationship is predominantly due to a single subject who was in a 23 year long relationship. When this subject is excluded there is no longer any significant relationship in the remaining group of 23 subjects (whose relationship lengths vary from 4 months to 6 years). There are two possibilities; either that this subject is an anomalous outlier, or there is a real effect that only becomes apparent in very long relationships. We cannot distinguish these two options with our sample. Perhaps a future study will resolve this issue.

**REFERENCES**

1. Hutton C, Josephs O, Stadler J, Featherstone E, Reid A, et al. (2010) The impact of physiological noise correction on fMRI at 7T. Proceedings of Joint Annual Meeting ISMRM-ESMRM, Stockholm, Sweden.

2. Glover GH, Li TQ, Ress D (2000) Image-based method for retrospective correction of physiological motion effects in fMRI: RETROICOR. Magn Reson Med 44: 162-167.

3. Birn RM, Diamond JB, Smith MA, Bandettini PA (2006) Separating respiratory-variation-related fluctuations from neuronal-activity-related fluctuations in fMRI. Neuroimage 31: 1536-1548.

4. Bartels A, Zeki S (2000) The neural basis of romantic love. Neuroreport 11: 3829-3834.
